# A standardized physician-modified endograft workflow utilizing the punch card technique and the Hungaroring reinforcement to treat complex abdominal aortic aneurysms

**DOI:** 10.1016/j.jvscit.2024.101649

**Published:** 2024-10-22

**Authors:** Csaba Csobay-Novák, Bendegúz Juhos, András Szentiványi, Ákos Bérczi, Artúr Hüttl, Péter Sótonyi

**Affiliations:** a Department of Interventional Radiology, Semmelweis Aortic Center, Heart and Vascular Center, Semmelweis University, Budapest, Hungary; b Department of Vascular and Endovascular Surgery, Semmelweis Aortic Center, Heart and Vascular Center, Semmelweis University, Budapest, Hungary

**Keywords:** Juxtarenal, Fenestrated, Physician-modified, Endovascular aortic repair

## Abstract

The physician-modified endograft technique is becoming widely accepted as an alternative to standard fenestrated endovascular aortic repair. We report a streamlined workflow based on a bifurcation endograft, using the punch card and the Hungaroring reinforcement, two novel additions to the armamentarium that might contribute to the safety and durability of such repairs. A cohort of 11 patients was treated with 43 vessels incorporated. The clinical success rate was 100%. No major adverse event or death was recorded at 30 days. The punch card and the novel reinforcement technique might improve the safety and durability of such repairs.

The physician-modified endograft (PMEG) technique was developed originally to treat symptomatic patients with aortic aneurysms requiring complex endovascular repair. Technical advancements, increasing experience, and promising early outcomes encouraged physicians to offer this therapy to patients in an elective setting as well, given its expedited nature that might be associated with a lower risk of interval rupture compared to those reported with standard fenestrated endovascular repair (FEVAR).^1^^,^^2^

The high heterogeneity of PMEG repairs across numerous centers limits the generalizability of single-center study results. To overcome this, worldwide registry data analyses, standardized techniques, and reporting are essential.^2^

We report our standardized approach and early outcome of juxta/pararenal abdominal aortic aneurysm treatment with the PMEG technique, using the punch card and the Hungaroring reinforcement based on a bifurcation endograft.

## Clinical management

A multidisciplinary aortic team of a tertiary vascular center evaluated patients with juxtarenal or pararenal aortic aneurysms >5.5 cm for repair. Expedited PMEG FEVAR has been offered for elective and urgent patients unfit for open repair within the framework of our ongoing observational trial (SE RKEB 75/2022). Emergent cases with hemodynamic instability requiring immediate repair were excluded. Informed consent was obtained in each case.

Anatomical suitability was evaluated on computed tomography angiography datasets. FEVAR was deemed possible if (1) an adequate proximal landing zone was present (≥3 cm long aortic segment with a 17- to 32-mm diameter, parallel walls, no significant calcification or mural thrombus, not higher than 5 cm above the midceliac artery), (2) the proximal segment of the renal-mesenteric arteries were accessible and suitable as a landing zone, and (3) adequate iliac landing zones and access vessels were present bilaterally.

## Technique

Planning was performed by the primary operator (C.C.N.) using 3Mensio Vascular software (Pie Medical Imaging, Maastricht, the Netherlands) following the very same principles as standard FEVARs. A full repair incorporating all renal-mesenteric arteries was aimed with a proximal edge positioned between 2 and 5 cm above the midceliac artery. The landing zone was maximized according to the ostium of the lowermost intercostal artery to be preserved with a 5-mm safety margin, resulting in a low supraceliac configuration.^3^ We used 10% to 20% oversize of the main body according to the landing zone diameter at zones 5 and 6. Fenestration diameters were 1 mm larger than the corresponding target artery diameter. Closed-ring reinforcements (Hungarorings) were prepared and sterilized as reported.^4^ To facilitate positioning, a punch card was also created and sterilized.^5^

**Fig 1:**
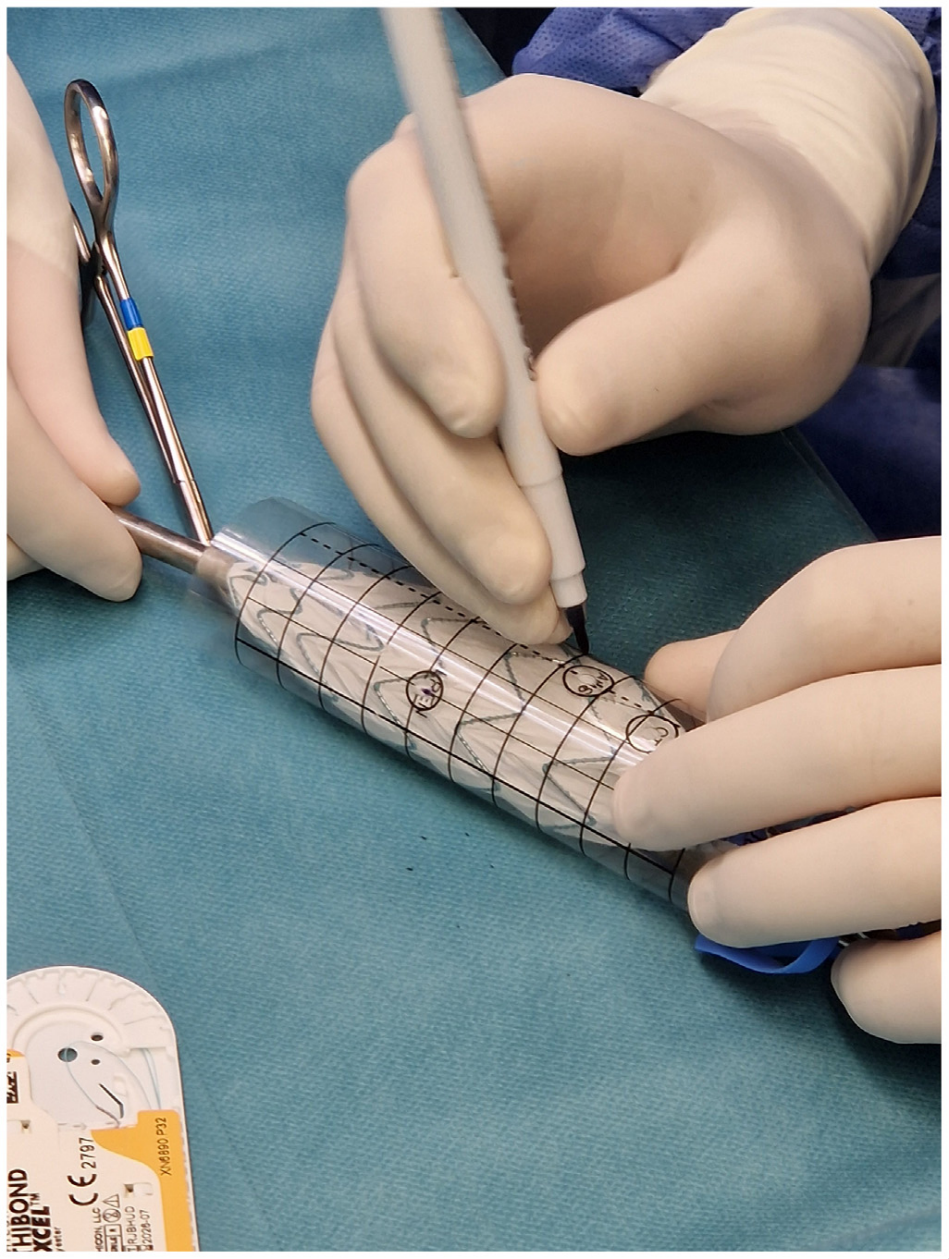
The punch card technique. A tube is formed from the punch card and the device is partially deployed into this tube. A position is searched when all fenestrations align over a strutless area of the device. Holes are marked with a sterile pen.

**Fig 2:**
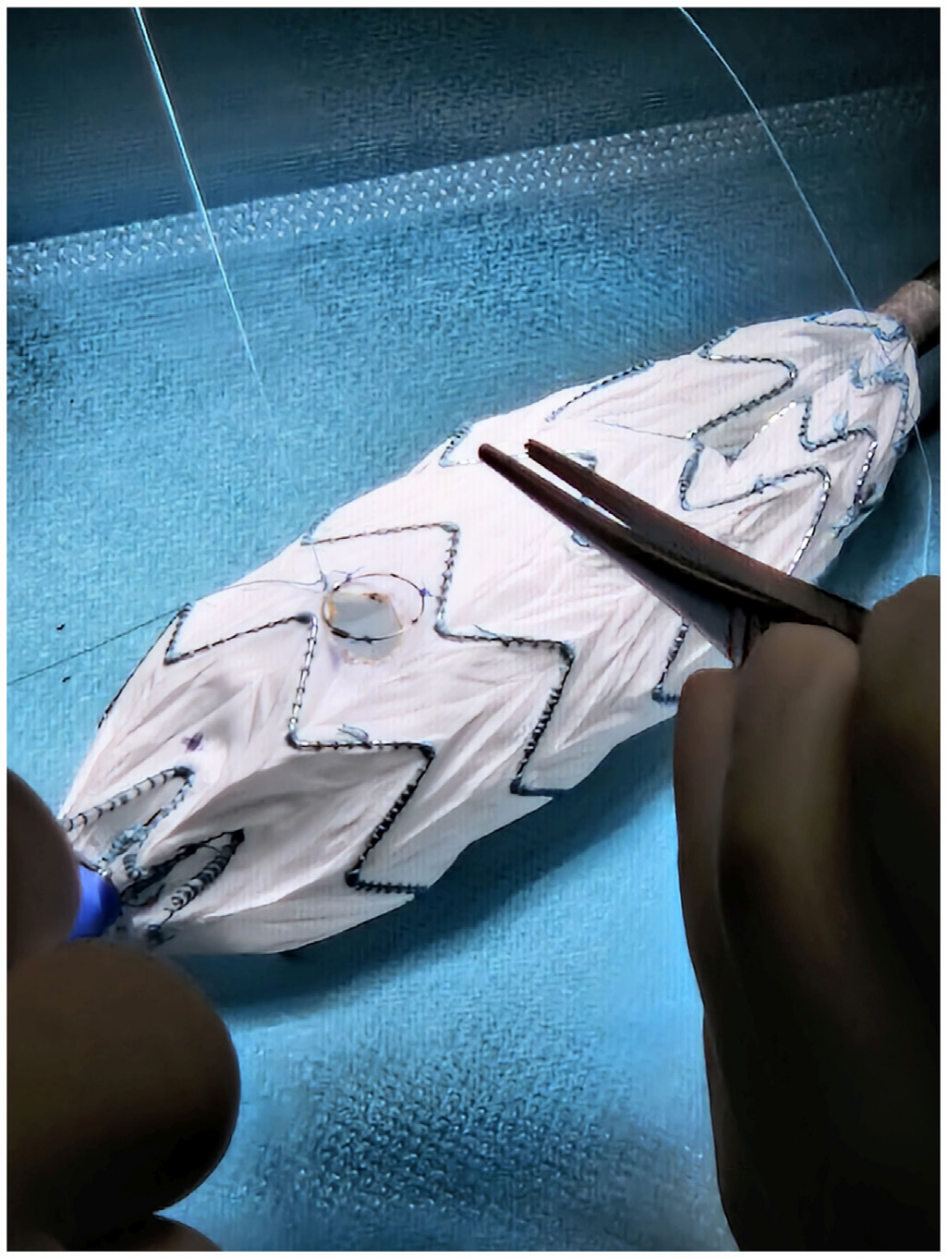
Reinforcement of the fenestrations with the Hungaroring.

**Fig 3:**
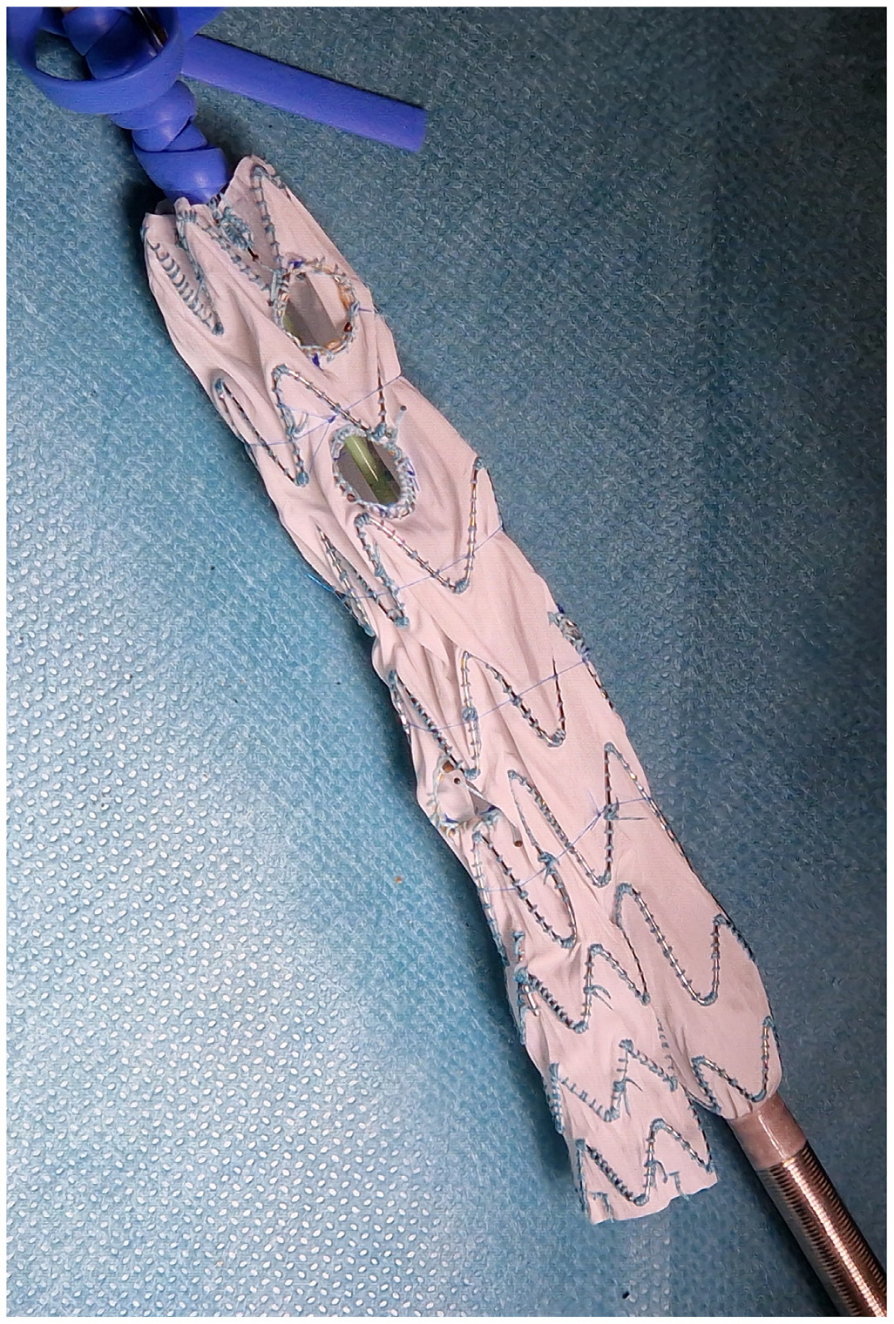
Circular diameter reducing ties are created over each stent row, aiming for a 50% overall diameter reduction.

**Fig 4:**
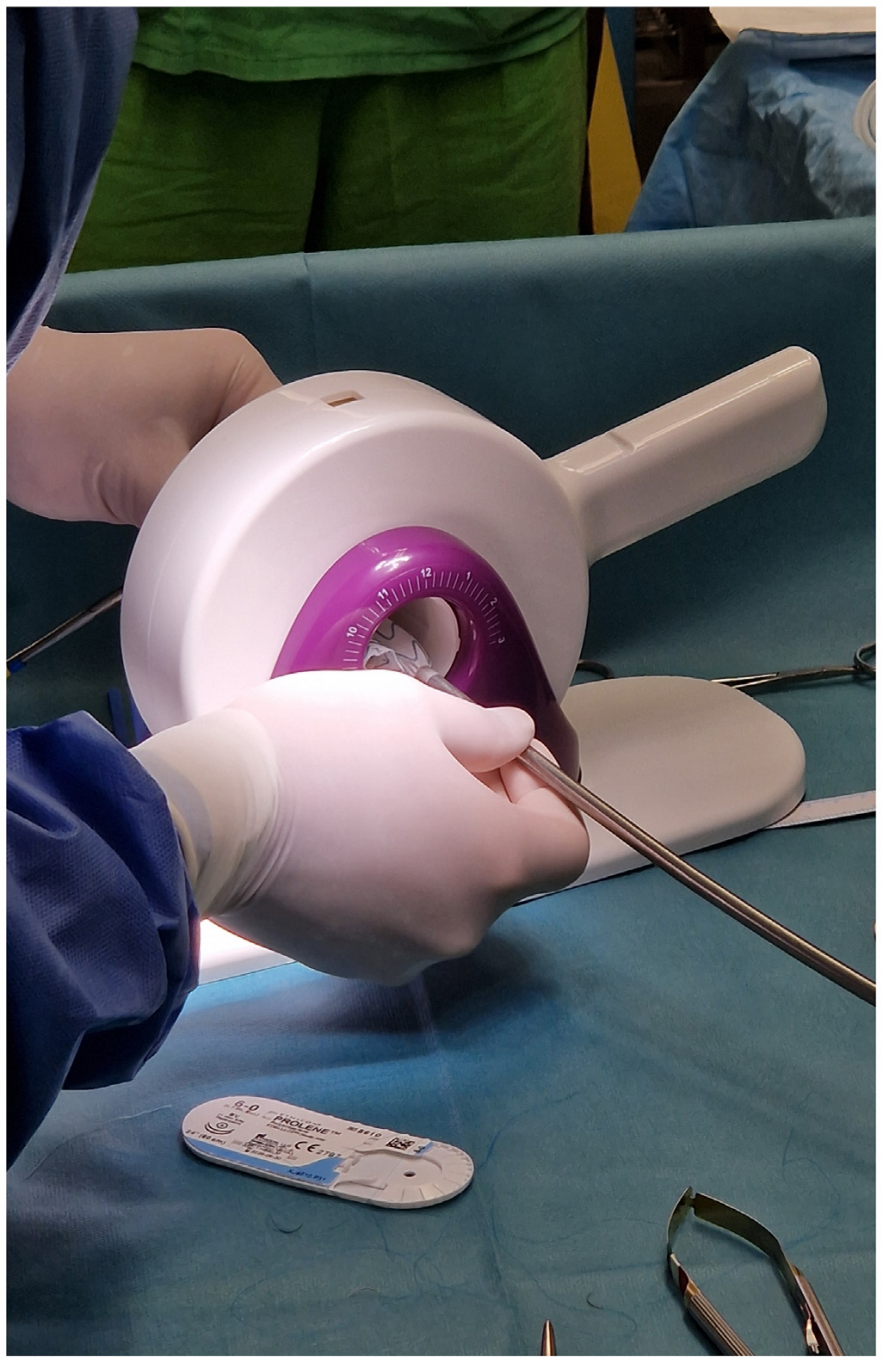
The physician-modified endograft is the reloaded into the delivery system using an aortic valve crimper.

Before and during the patient's preparation for general anesthesia, the bifurcation main body (Treo; Terumo Aortic, Inchinnan, UK) was partially deployed into the tube formed by the punch card (Fig 1).^5^ After reaching an optimal configuration, positions were marked through the punch card holes with a sterile pen, and then holes were created on the prosthesis using a sterile cautery.^5^ Each fenestration was then reinforced with an appropriately sized Hungaroring using continuous locking polyester sutures (5-0 Ethibond; Ethicon Inc., Raritan, NJ) (Fig 2). Circular diameter-reducing ties were then positioned around each stent row of the main trunk using a monofilament suture (6-0 Prolene; Ethicon Inc.) to achieve approximately a 50% diameter reduction (Fig 3). The device was then reloaded into the delivery system with a transcatheter aortic valve repair crimper (Val-de-Crimp; Meril Life Sciences, Vapi, Gujarat, India) (Fig 4).

The prepared device was then inserted into a 20F 33 cm transfemoral sheath (DrySeal; Gore Medical, Flagstaff, AZ). After proper orientation of the fenestrations, the device was slowly deployed until the contralateral gate. The fenestrated main body was cannulated, followed by the insertion of a 12F 45 cm transfemoral sheath (DrySeal; Gore Medical). A 260 cm Rosen wire (Cook Medical Inc., Bloomington, IN) was positioned in each target artery, after which the main body delivery system was removed. The diameter-reducing ties were ruptured with a compliant balloon (Reliant; Medtronic plc, Dublin, Ireland), and the 20F sheath was pulled down below the hypogastric ostium. Bridging stents were then sequentially deployed and flared, followed by an iliac limb extension bilaterally. Completion anteroposterior angiography and unenhanced cone-beam computed tomography was performed to evaluate device integrity and conformation. The patient was extubated on the table, a rapid neuro-check was performed in the operating room to evaluate the need for a therapeutic cerebrospinal fluid drainage and the patient was then transferred back to the vascular surgery ward. A predischarge computed tomography angiography is performed, followed by scheduled follow-ups similar to standard FEVAR cases.

## Results

Eleven patients (8 males; 76 ± 5 years of age) underwent PMEG FEVAR with a bifurcation graft during a 10-month period. A total of 43 vessels were incorporated, resulting in an average of 3.9 fenestrations per patient. Patient characteristics and procedural data are shown in the Table.

**Table:** Baseline characteristics and procedural data (n = 11)

Characteristics	No. (%) or mean ± standard deviation
Patient characteristics	
Male sex	8 (72.7)
Age, years	76 ± 5
Body mass index, kg/m^2^	28 ± 6
Diameter of aneurysm, mm	71 ± 7
Symptomatic aneurysm	2 (18.2)
Procedural data	
Preparation time, minutes	63 ± 13
Fluoro time, minutes	82 ± 39
Dose area product, μGym^2^	182 ± 162
Total radiation dose, mGy	2065 ± 1866
Contrast dose, mL	181 ± 52
30-Day outcomes	
Reintervention	1 (9.1)
Length of stay, days	4.5 ± 1.5
Endoleak	2 (18.2)
Major adverse event	0 (0)
Death	0 (0)
Technical success per patient	10 (90.9)
Technical success per vessel	42 (97.7)
Clinical success	11 (100)

Intensive care unit observation was not needed. A single technical failure was observed associated to an accessory renal. The lost artery was supposed to be the fifth incorporated vessel with a diameter of 3 mm. Neither decline in renal function nor endoleak was detected in association with the sacrificed accessory renal and the unstented fenestration. No sign of spinal cord ischemia was detected. Two type II endoleaks were detected, neither of which required reintervention. No type I or III endoleaks were observed. A single in-hospital reintervention was performed in a patient who developed new-onset severe claudication associated with an access-related common femoral stenosis, resolved by a transbrachial balloon angioplasty.

## Discussion

We report the initial patient cohort of our streamlined workflow for juxta/pararenal aortic aneurysms with PMEG FEVAR using a bifurcation endograft. This report represents the first implants that were prepared using the punch card technique and the first patients treated with PMEGs that have closed-ring reinforcements, similar to the reinforcement of custom-made devices.

With the help of this technique, three-dimensional printing can be omitted from the workflow that is necessary if cylindrical or anatomical three-dimensional models are being used for positioning.^6^ Templates can be created and sterilized in advance, and can be transformed to punch cards with a scalpel on the backtable, making it an ideal tool for urgent cases.

The punch card technique provides a fast and accurate way of positioning the fenestrations in a configuration where no struts cross any fenestrations. Eliminating the need to manipulate the skeleton of the aortic components may improve the long-term durability of the repair.^7^^,^^8^ This technique can be used with any type of aortic implant. Our approach with the punch card is significantly faster compared with manual measurements reported recently by Piazza et al.^9^

A closed-ring reinforcement of the fenestrations allowed us to use oversized balloons with high pressure for flaring in a similar manner that is usual with standard FEVARs, confirming previous in vitro findings.^10^ The design is very similar to custom-made device fenestrations. This might convert to a durability benefit compared with other reinforcement techniques.

The use of a bifurcation as a PMEG might be advantageous by excluding the risk of junctional separation.^11^

## Conclusions

We report a streamlined workflow for juxta/pararenal abdominal aneurysm treatment using the PMEG technique and a bifurcation design. In addition to previous reports, the punch card technique improves precision and speed, while the Hungaroring reinforcement hopefully adds durability to our repairs. Standardization of the PMEG technique is essential to provide a basis of further studies regarding the long-term outcome of such repairs.

## Funding

This study was supported by the National Research, Development and Innovation Office of Hungary (2024-2.1.2-EKÖP-KDP-2024-00002).

## Disclosures

C.C.N. is a trainer, proctor, and speaker for Terumo Aortic, Cook, Medtronic, and W. L. Gore & Associates.
